# Not Just Pigments: The Significance of Carotenoid Metabolism in Crab Aquaculture

**DOI:** 10.1155/anu/9060019

**Published:** 2026-11-15

**Authors:** Saeed Ullah, Muhammad Nadeem Khan, Jiangtong Han, Gongyuan Wei, Chonglong Wang

**Affiliations:** ^1^ School of Life Sciences Soochow University Suzhou 215123 China scu.edu.tw; ^2^ Institute of Agricultural Biotechnology and Ecological Research Soochow University Suzhou 215123 China scu.edu.tw

**Keywords:** carotenoids, crab aquaculture, dietary supplements, metabolic engineering, pigmentation

## Abstract

In crab aquaculture, carotenoids exert indispensable and multifaceted functions that extend well beyond pigmentation, influencing hepatopancreas and ovary development. As crabs cannot synthesize carotenoids de novo, these compounds must be supplied through the diet. This review summarizes current knowledge of the physiological significance, metabolic pathways and functional mechanisms of carotenoids in crabs. It also examines the complex processes of carotenoid absorption, transport, tissue‐specific deposition and enzymatic transformations, including cleavage and isomerization, while highlighting the significant gaps that remain in our understanding of the regulatory networks governing these processes. This review is expected to deepen the recognition of carotenoids as multifunctional health factors, to promote the use of synthetic biology and metabolic engineering in the development of microbial platforms for high‐value carotenoids such as astaxanthin, and to formulate precise dietary strategies that improve crab cultures.

## 1. Introduction

Aquaculture is the fastest‐growing food sector and offers an important means of meeting the growing demand for food in coastal communities [1]. Crustaceans, particularly the decapods, such as crabs, lobsters, and shrimp, are a diverse and commercially important group [2]. Crabs are cultivated for food, processed on a large scale, and favored as commercial seafood. According to FAO data, the Chinese mitten crab (*Eriocheir sinensis*) yielded ~815,000 tonnes in 2022, accounting for 6.4% of the crustacean aquaculture production, ranked the third among cultured species [3]. Many aquatic organisms display extraordinary colors as a result of the accumulation of dietary carotenoids [4]. These pigments determine coloration, growth, and immune performance and thereby enhance market value [5, 6]. Sustainable and profitable culture of the *E. sinensis* is constrained by inconsistent, sex‐ and tissue‐specific carotenoid pigmentation of the carapace, ovary, and hepatopancreas, which complicates dietary strategies aimed at uniform commercial coloration [7, 8].

Pigmentation in crabs is recognized as an important quality indicator affecting market value and consumer acceptance. Crabs lack the full metabolic capability to synthesize carotenoids de novo; instead, pigmentation depends on dietary intake of carotenoids from feed and their surroundings [9]. Besides, the accumulation of carotenoids in crabs makes these aquatic animals an important source of natural carotenoids for other animals and humans [7]. Carotenoids including astaxanthin, *β*‐carotene, lycopene, lutein, and zeaxanthin are often used as feed supplements in crab aquaculture. In crustaceans, carotenoids confer multiple physiological benefits, including provitamin A activity, enhanced coloration and growth performance, antioxidant capacity, disease resistance, immune modulation, and tolerance to environmental stressors [10]. As a source of provitamin A, carotenoids improve the productivity of aquatic animals in aquaculture [11]. They also act as biological antioxidants to protect tissues from the damaging effects of free radicals and enhance immune system function [6, 12]. Crabs remain persistently vulnerable to environmental stress and diseases that undermine survival, growth, and reproduction [13]. Synthetic carotenoids still dominate aquafeed formulations because of their lower production cost. However, consumer demand is increasingly shifting toward natural carotenoids owing to health and environmental concerns associated with synthetic pigments. There are key gaps in understanding the bioavailability, chemical forms, transport mechanisms, and tissue‐specific deposition of carotenoids in crabs, as well as poorly defined stage‐specific nutritional requirements, which constrain the rational design of pigmentation and nutritional strategies for the aquaculture industry.

Formulating nutritionally adequate feed is an essential prerequisite for normal crab growth [4]; in particular, appropriate and balanced diets containing carotenoids must be fed to reared larvae to achieve optimal survival and development. Although both natural and synthetic carotenoids serve as pigment sources for crustacean species at various level [2, 8]. The crab aquaculture industry aims to find eco‐friendly, cost‐efficient carotenoids that enhance coloration and consumer acceptability while improving survival and growth [14]. This review highlights the significance of carotenoids in crab aquaculture, including their use as dietary supplements to improve coloration, growth and reproductive performance, antioxidant capacity, and innate immunity. In the light of advances in metabolic engineering and synthetic biology, it also outlines the prospects and future direction for microbial production of carotenoids in crab aquaculture.

## 2. Carotenoids in Crab Aquaculture

Carotenoids are a large number of natural pigments found in organisms as diverse as microalgae, bacteria, and plants [15–17]. Generally, eight isoprene units of carotenoids are covalently attached to form a polyene chain with conjugated *π*‐*π* bond (Figure 1a) that endows carotenoids with photochemical properties to quench singlet‐oxygen and scavenge free radicals [18]. In aquaculture, carotenoids have been shown to improve the egg quality, offspring survival, and growth of crustaceans [7, 11]. Dietary astaxanthin combined with docosahexaenoic acid had a positive effect on ovarian development and improved pigmentation in adult female *E. sinensis* [19]. In *Portunus trituberculatus*, by contrast, astaxanthin supplementation enhanced lipid utilization, egg diameter, and oxidative stress resistance [20]. Carotenoids fall into two main classes, xanthophylls (e.g., astaxanthin, zeaxanthin, canthaxanthin, and lutein) and carotenes (e.g., lycopene, *α*‐carotene, and *β*‐carotene), according to the presence or absence of oxygen‐containing functional groups [21, 22]. Xanthophylls are thus slightly more hydrophilic than carotenes. Animals, including crabs, have no de novo biosynthesis pathway of carotenoids, which must be acquired from dietary sources. The intake of lipophilic carotenoids follows transport and metabolic mechanisms similar to those of lipids in the aqueous environment of the body and is finally accumulated within various tissues and organs [23, 24]. Nutritional composition governs ovarian development, offspring viability, growth, and flesh quality in mud crabs [25]. Carotenoids may play a functional role in crustacean reproduction, though direct evidence linking carotenoid supplementation to fertilization, hatching, and larval survival in crab species remains limited.

**Figure 1:**
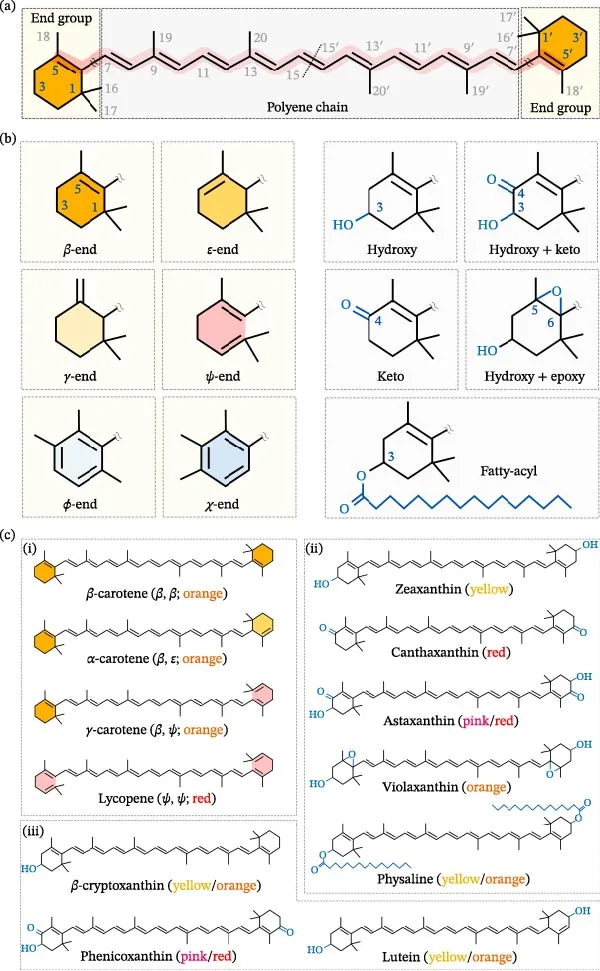
The chemical structure of carotenoids. (a) The basic carotenoid skeleton. Carotenoids are composed of one polyene chain and two end groups. The conjugated polyene chain is shadowed in pink. The C40 carotenoids are generally partitioned with two asymmetric C20 units, and carbon numbering follows International Union of Pure and Applied Chemistry rules. (b) The end groups and their modification. Hydroxylation and ketolation of the end groups typically occur at the C_3_ and C_4_, respectively. The epoxidation is at the double bond between the 5th and 6th carbons. (c) The representative carotenoids and the categories. Carotenoids are categorized into carotenes (i) and xanthophylls, which include the symmetric (ii) and asymmetric (iii) products according to the modification position.

### 2.1. Carotenoids Sources and Their Biosynthesis

Carotenoids are widely distributed in plant algae, bacteria, and fungi. Microalgae are recognized as a natural source of carotenoids, although the production is not yet sufficiently cost‐effective to compete with traditional chemical synthetic methods [15]. The biosynthetic pathways of carotenoids differ among various organisms and are species‐specific. Briefly, carotenoid biosynthesis includes the formation of geranylgeranyl diphosphate (GGPP, C20) precursor and modification of colorless phytoene (C40). The GGPP formation has been summarized elsewhere [26], and this review mainly describes the phytoene modification, including desaturation, cyclization, hydroxylation, ketolation, epoxidation, and isomerization in the modification process. The linear phytoene undergoes four desaturation steps through sequential reactions catalyzed by phytoene desaturase, and *ξ*‐carotene desaturase, and carotenoid isomerase, ultimately generating red‐colored lycopene in plants and algae [27, 28]. However, most carotenogenic prokaryotes and fungi have a sole phytoene desaturase, such as *Pantoea ananatis* CrtI, taking over the four‐step reaction for lycopene generation [29]. The cyclization of linear lycopene is generally recognized as an important divergence point for carotenoid biosynthesis, which gives rise to the orange *β*‐carotene with two *β*‐end groups by lycopene *β*‐cyclase (LCYb) or *α*‐carotene with *β*‐ and *ε*‐ end groups formed by LCYb and lycopene ε‐cyclase (Figure 1b, c‐i) [30]. Carotene hydroxylase, ketolase, and epoxidase then modify both ionone rings to generate symmetric xanthophylls, such as zeaxanthin, canthaxanthin, astaxanthin, and violaxanthin (Figure 1c‐ii) [18, 31]. Such modifications can occur in different rings or at different carbon positions, yielding asymmetric xanthophylls (Figure 1c‐iii), which significantly improves the diversity of carotenoids. This structural divergence is fundamental to understanding carotenoid metabolism in crabs.

Some microorganism species (e.g., *Paracoccus carotinifaciens*, *Phaffia rhodozyma*, *Haematococcus pluvialis*, *Chlorella vulgaris*, *Chlorococcum* sp., and *Chlorella zofingiensis*) represent promising alternative sources of astaxanthin, widely used as a feed ingredient for salmon, trout, shrimp, and crayfish [32–34]. *H. pluvialis*‐derived astaxanthin is the most extensively characterized source of bioavailable astaxanthin for culturing *Scylla paramamosain* and *E. sinensis* [35, 36]. Its bioavailability, however, is governed by chemical form (e.g., esterified or free astaxanthin), transport proteins, and species‐specific physiology, as well as intestinal absorption efficiency and tissue storage capacity [37]. Elucidating these mechanisms is essential for rationally designing feed formulations that maximize astaxanthin bioavailability in cultured species.

### 2.2. Storage and Absorption of Carotenoids in Crabs

Crab coloration is the most important sensory quality indicator and largely determines consumer acceptability. For instance, crab products of *E. sinensis*, *P*. *trituberculatus*, and *Scylla olivacea* are preferred when hepatopancreas and ovaries are highly colored [7, 38–40]. Carotenoids and crustacyanin play important roles in crab coloration, whereas carotenoids cannot be synthesized de novo in crabs. Diet is therefore the determining factor for coloration in cultured crabs [41]. With carotenoid uptake, hepatopancreas and ovaries are developed and matured, manifesting yellow, orange, or red coloration (Figure 2a). Carotenoids are dominantly accumulated in carapaces, hepatopancreas, and ovaries. *β*‐Carotene and xanthophylls of lutein, zeaxanthin, canthaxanthin, and astaxanthin are the main accumulated species in *E. sinensis* (Figure 2b) [43, 44]. The distribution of carotenoids varies among crab tissues, which are also influenced by diets, individual differences, and environment. In a preliminary survey of *E. sinensis* (*n* = 60 females and 60 males), the mean carotenoid content of the carapace was much higher in females (59.10 ± 2.29 mg/kg dry weight) than that in males (44.72 ± 2.43 mg/kg dry weight), whereas the opposite was found in hepatopancreas (123.22 ± 14.16 vs. 237.49 ± 29.05 mg/kg dry weight). The mean content reaches 554.70 ± 12.97 mg/kg dry weight in ovaries (left panel, Figure 2c). With respect to carotenoid composition, carapaces and ovaries tend to accumulate xanthophyll species, and hepatopancreas accumulates proportionally more carotenes (right panel, Figure 2c). It should be noted that each tissue has a distinct capacity to deposit different carotenoids, which poses a challenge for crab feed preparation [42].

**Figure 2:**
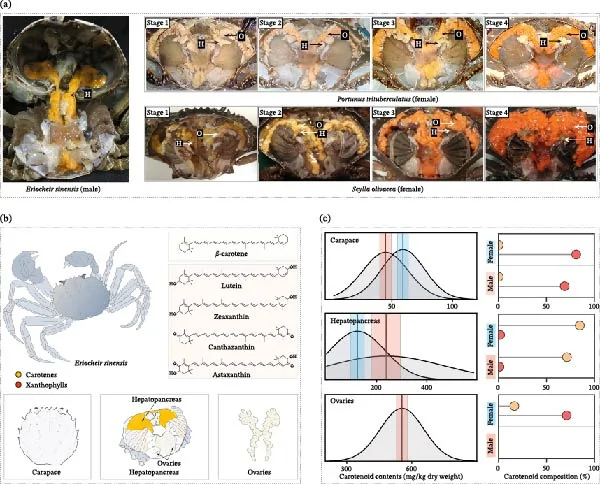
The distribution and composition of carotenoids in crabs. (a) Morphological images of crab tissues accumulated carotenoids. The images were reproduced from the literature [38–40]. (b) The main accumulated carotenoids and the deposit tissues in *E. sinensis*. (c) The carotenoid contents and composition in the tissues of *E. sinensis*. The plots were drawn with the data from the literature [42]. The red and blue shadowed boxes indicate the total carotenoid contents in the tissue of female and male crabs. The red and orange lollipops represent the composition of carotenes and xanthophylls.

Upon ingestion, dietary carotenoids are incorporated into micelles within the stomach and transported by means of chylomicrons to the hemolymph, where lipoproteins such as crustacyanin are thought to bind astaxanthin for storage and utilization in organs (Figure 3a) [23]. In this process, lipophilic carotenoids are probably taken up by absorptive cells in microvilli through direct diffusion and transporters of SR‐B1, CD36, or NPC1 [45]; how carotenoids enter the hemolymph remains to be elucidated (upper panel, Figure 3b). SR‐B1 has been shown to be essential for carotenoid‐based coloration in zebrafish and common carp [46, 47]. Similarly, transcriptomic analysis linked CD36 upregulation to improved dietary astaxanthin absorption in Atlantic salmon [48], while chenodeoxycholic acid supplementation was found to upregulate NPC1 and facilitate cholesterol transport in shrimp [49].

**Figure 3:**
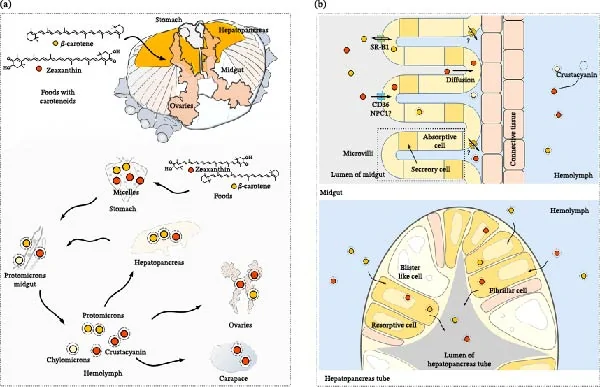
The possible transportation of carotenoids in crab. (a) Possible digestive route of the representative carotenoids, *β*‐carotene and zeaxanthin. Carotenoids in food are subject to digestion in stomach and form micelles, which are probably further absorbed in midgut and further transported to hemolymph and hepatopancreas via protomicrons. Carotenoid molecules can also be transformed in the hepatopancreas within the physiological requirements. (b) Carotenoid absorption in midgut and hepatopancreas. Carotenoids are absorbed into midgut and hepatopancreas via carrier proteins, and most of the transformation occurs in the lumen of hepatopancreas tubules. Abbreviations: SR‐B1, scavenger receptor class B type 1; CD36, cluster of differentiation 36; NPC1, Niemann‐Pick C1.

The hepatopancreas is regarded as the major internal organ for storage and assimilation of carotenoids for tissue growth in crustaceans [6, 7]. Although resorptive (R) cells participate extensively in nutrient absorption and lipid metabolism, their specific role in carotenoid trafficking remains unclear. R cells probably transfer carotenoids from hemolymph to lumen of the hepatopancreas tubule, where they are further hydroxylated, oxidized, or cleaved, producing derivatives of great importance for molting and maturation. In *E. sinensis*, carotenoids accumulated in the hepatopancreas undergo metabolic transformations, including *β*‐carotene cleavage mediated by carotenoid cleavage oxygenases such as BCO1‐like EsCCO3 and NinaB‐like EsCCO6, generating retinoid‐related metabolites [50, 51]. Carotenoid absorption rates in the hepatopancreas are principally limited by the saturation response of transporters; however, there is limited information on carotenoid trafficking in hepatopancreas. A worthwhile direction is to mine the possible transporter candidates from the steroidogenic acute regulatory domain‐containing (STARD) proteins, which usually associate with lipid transfer in the binding and transport of cholesterol and steroid hormones. Based on expression differences and molecular docking analysis, STARD3 and STARD11 have been identified as putative intracellular lipid and carotenoid trafficking proteins in *E. sinensis* [52]. Interestingly, STARD3 is capable of binding lutein and has been identified as a carotenoid‐binding protein in *Bombyx mori* [53]. STARD11 is essential for sphingomyelin synthesis and is suggested to maintain membrane stability of digestive tract and lipid balance [54]. Lipophilic carotenoids tend to deposit in lipids or membranes, like cholesterol (Figure 4a), and it is reasonable to speculate that carotenoids share the same trafficking route as cholesterols [56]. Additional evidence is that dietary cholesterol supplementation significantly improves tissue deposition of carotenoids [57].

**Figure 4:**
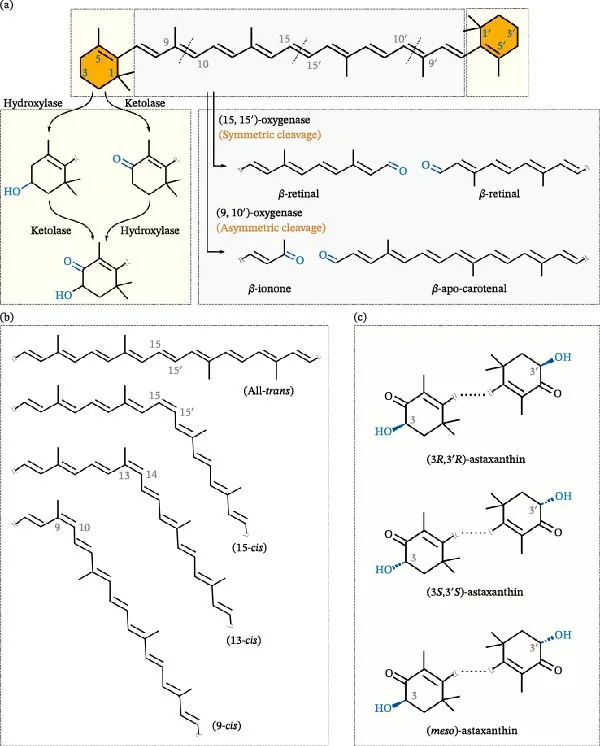
The storage of carotenoids in crabs. (a) The possible deposition model of carotenoids. (b) The carapace morphologies associated with crustacyanin and the crustacyanin structures. The carapace imaging was retrieved from the literature [55]. The crystal structure of *β*‐crustacyanin is rendered from the collection 1GKA. It is composed of CRTC‐A2 and CRTC‐A3, the two apoproteins of CRTC‐A and CRTC‐C subgroups. Eight *β*‐crustacyanin units assemble into *α*‐crustacyanin. (c) Phylogenetic analysis of crustacyanins. The protein sequences were obtained from UniProt. The circles and squares represent the CRTC‐A and CRTC‐C subgroups. The crustacyanins from one species are shadowed with the same color.

Carotenoids, especially astaxanthin, can be bound to crustacyanin for shell coloration. The formation of carotenoid‐crustacyanin complex shifts the absorption spectrum of the carotenoids toward longer wavelengths (a bathochromic shift), so that the complex appears blue rather than red [58, 59]. Astaxanthin transport is closely linked to carapace coloration (Figure 4b). Denaturation of crustacyanin during cooking releases the encaged carotenoids, turning the carapace red. X‐ray crystallography (PDB: 1GKA) investigations revealed that two astaxanthin molecules are encapsulated within heterodimeric *β*‐crustacyanin in the lobster *Homarus gammarus* (Figure 4b) [60]. To date, five typical crustacyanin isoforms (C_1_, C_2_, A_1_, A_2_, and A_3_) have been elucidated from the lobster [61], and *β*‐crustacyanin is generally composed of two apoproteins (~20 kDa) from different subtypes CRTC‐A (A_2_ and A_3_) and CRTC‐C (C_1_, C_2_, and A_1_). These reconstituted *β*‐crustacyanins modify the color of the carotenoid chromophore and endow a color range of yellow to red [61–63]. Eight *β*‐crustacyanin units are then assembled into *α*‐crustacyanin harboring a total of 16 astaxanthin molecules [64]. A few crustacyanin genes have been identified in crabs, and four crustacyanin analogs exhibit relatively low similarity to those of shrimp (Figure 4c). It was speculated that crustacyanins transport carotenoids from hemolymph to carapace, while the bona fide crustacyanin genes determining coloration remain to be identified. Some solute carriers and the low‐density lipoprotein receptor may also be involved in carotenoid transport [65].

### 2.3. Carotenoid Metabolism in Crabs

Crabs, like other crustaceans, are incapable of de novo carotenoid biosynthesis and must acquire these pigments exogenously, primarily through dietary sources such as algae and zooplankton. Once ingested, carotenoids are absorbed and metabolized in the hepatopancreas and then either transformed or transported directly for deposition in the epidermis and other tissues [10, 66]. This metabolic conversion is not a single‐step event but comprises three interconnected processes: oxidation and cleavage, isomerization, and stereoisomerization.

#### 2.3.1. Carotenoid Oxidation and Cleavage

The conversion of *β*‐carotene into astaxanthin is central to carotenoid metabolism in crabs. Absorbed dicyclic *β*‐carotene probably undergoes hydroxylation and/or ketolation, yielding *β*‐cryptoxanthin, zeaxanthin, echinenone, canthaxanthin, and adonixanthin, as intermediates in the astaxanthin biosynthesis [18, 67]. These modifications of *β*‐end ring occur specifically through the addition of keto group at C_4_ (C_4_′) positions and of hydroxyl groups at the C_3_ (C_3_′) positions (Figure 5a). These *β*‐carotene ketolases and hydroxylases are common in carotenogenic plants, algae, cyanobacteria, and phototrophic bacteria [21, 29], but both have rarely been identified in arthropods, including crabs [68]. Interestingly, some cytochrome P450 enzymes, such as CYP389B1 in *Tetranychus urticae* and *Taeniopygia guttata* and CYP384A1 in *T. kanzawai*, regulate pigmentation of their tissues [69–71], suggesting that a similar conversion of *β*‐carotene to ketocarotenoids may be present in crabs.

**Figure 5:**
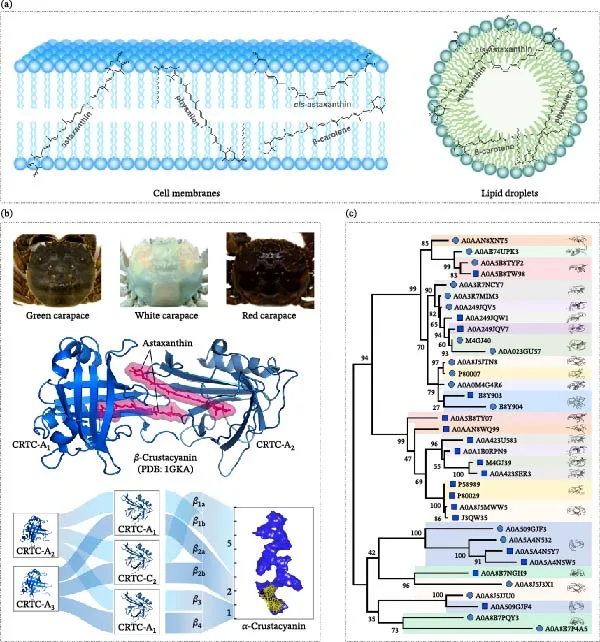
The transformation of carotenoids in crab. (a) The modification and cleavage of *β*‐carotene. End group modification is catalyzed by hydroxylase and ketolase at C_3_ or/and C_4_, and the cleavage of polyene chain occurs at the C_15_ ⚌ C_15_′ and C_9_ ⚌ C_10_ double bonds by distinct oxygenases. (b) The *trans*‐*cis* isomerization of polyene chain. (c) The stereoisomerization of astaxanthin.

Another main metabolic process of *β*‐carotene is the oxidative cleavage in the polyene chain by 15,15′‐*β*‐carotene oxygenase (BCO1) into two molecules of retinal (Figure 5a), which can then be further oxidized irreversibly to retinoic acid or reduced reversibly to retinol [72]. Carotenoid isomerooxygenase (NinaB), first identified in *Drosophila*, catalyzes the oxidative cleavage at C_15_ ⚌ C_15′_ bond and the *trans*‐to‐*cis* isomerization of the C_9_ ⚌ C_10_ bond of retinoids [51, 55, 73]. The asymmetric cleavage via 9,10‐*β*‐carotene oxygenase (BCO2) results in the formation of *β*‐ionone and *β*‐apo‐10′‐carotenal [74, 75]. In *E. sinensis*, six carotenoid cleavage oxygenases (EsCCO1–6) were mined from the assembled genome (ASM2467909v1), and they showed distinct expression patterns within various tissues [76]. Further analysis revealed that EsCCO3 and EsCCO6 are BCO1‐like and NinaB‐like enzymes, respectively, whose dysfunction can lead to a significant accumulation of *β*‐carotene in the hepatopancreas [51], while BCO2 was predominantly expressed in the hindgut, with significantly higher expression levels in females than in males [74].

#### 2.3.2. Carotenoid Isomerization and Stereoisomeric Carotenoids

The second metabolic process would be carotenoid isomerization (Figure 5b). All‐*trans* carotenoids (e.g., all‐*trans* astaxanthin) are more thermodynamically stable owing to the lower steric hindrance than the *cis*‐configurations and are the predominant geometric form in nature. Although 9 double bonds of the polyene chain could generate 271 geometric *cis*‐isomers, only a few *cis*‐configurations (9‐*cis*, 13‐*cis*, and 15‐*cis*) occur naturally [77]. Such structural *trans*‐to‐*cis* transformations of the polyene chain result in different electronic, vibrational, and chemical properties of the carotenoids, conferring greater biological activity [78, 79]. Both 9‐*cis* and 13‐*cis* astaxanthin showed a higher protective effect than all‐*trans* astaxanthin against oxidative stress [80]. Feeding with 9‐*cis* astaxanthin extended the median lifespan of *Caenorhabditis elegans* [81].

Moreover, the hydroxylation at the C_3_ (C_3_′) position of the *β*‐ionone ring would generate (3*R*,3′*R*) and (3*S*,3′*S*) enantiomers as well as a *meso* (3*R*,3′*S*) form (Figure 5c). The formation of these optical isomers varies across species, likely due to the hydroxylase specificity [82]. For example, (3*S*,3′*S*)‐astaxanthin is the predominant product in salmon, whereas shrimp and crabs contain a mixture of all three isomers [83, 84]. A feeding experiment with astaxanthin stereoisomers indicated that the composition of astaxanthin in crabs does not entirely match that of the diet [85], suggesting a sophisticated transformation mechanism of carotenoids. However, direct measurements of intestinal transport and in vivo isomerase activity are still lacking; it is likely that a selective absorption and isomerization process exists in crabs, ensuring their combined effect for biological adaptation. A worthwhile observation was that different astaxanthin stereoisomers trigger distinct gene expression associated with oxidative stress [86].

#### 2.3.3. Carotenoid‐Derived Retinoids

Vivid carapace coloration and paradigmatic antioxidant activity are the most noticeable features of carotenoids in aquaculture, while other functions remain to be elucidated. A transcriptomic analysis study suggested dietary carotenoids could also enhance the immune capability of *E. sinensis* via the pathways of antimicrobial peptides, prophenoloxidase system, pattern recognition receptors, integrins, and apoptosis [87]. The best‐understood mechanisms of carotenoid action in ovary development, cell growth, antioxidant defense, and immune response center on retinoids derived from a symmetrical cleavage of *β*‐carotene (Figure 6). Retinal is the direct product of *β*‐carotene cleavage by BCO1 in the hepatopancreas [51, 76, 88], and further oxidation by retinal dehydrogenase converts retinal to retinoic acid. The asymmetric cleavage products by BCO2 and the invertebrate‐type NinaB represent parallel entry points into the same retinoid pool. Thus, carotenoid‐derived retinoids provide a direct mechanistic link between dietary carotenoids and RAR/RXRs‐mediated regulation of ovarian development and lipid metabolism.

**Figure 6:**
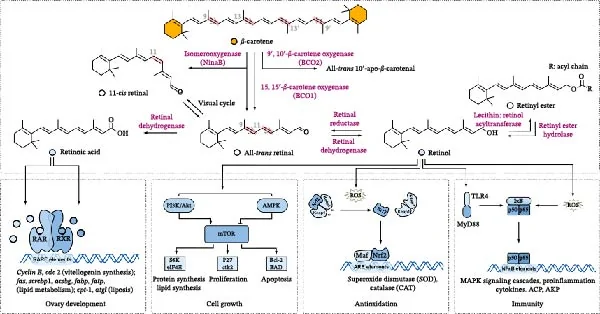
The retinoid homeostasis in crab and its possible regulatory function. The symmetrically cleaved product of *β*‐carotene is further oxidized to retinoic acid or reduced to retinol, which can regulate transcript factors and signal pathways for ovary development, cell growth, antioxidation and immune responses. The esterified form can serve as a reservoir that buffers cellular retinol level. In addition, *β*‐carotene can be cleaved to form 11‐*cis*‐retinal or *β*‐apo‐10′‐carotenal, which are also involved in retinoid homeostasis. Abbreviations: ACP, acid phosphatase; AKP, alkaline phosphatase; AMPK, adenosine 5′‐monophosphate (AMP)‐activated protein kinase; BCO1, 15,15′‐*β*‐carotene oxygenase; BCO2, 9,10‐*β*‐carotene oxygenase; MAPK, mitogen‐activated protein kinase; mTOR, mechanistic target of rapamycin; NinaB, isomerooxygenase; Nrf2, nuclear factor E2‐related factor 2; RARs, retinoic acid receptors; ROS, reactive oxygen species; RXRs, retinoid X receptors.

All‐*trans* retinoic acid, the main active metabolite of vitamin A, binds and activates RARs, which act as heterodimers with RXRs [89]. Differential RAR/RXR heterodimer combinations shape gene expression outcomes during ovarian development including vitellogenin synthesis [90]. In *S. paramamosain*, ovarian follicular RXR expression increased during maturation, and RXR and vitellogenin genes were coinducible in ovarian explant culture, indicating that RXR acts as a direct upstream regulator of the vitellogenin gene [91]. Similarly, the expression of RXR and key genes involved in ovarian development was upregulated when Chinese mitten crabs were fed with all‐*trans* retinoic acid [92]. These studies identify vitellogenin, EcR, and E75 as specific RAR/RXR‐regulated targets underlying carotenoid‐driven ovarian development in crabs, providing concrete molecular support for RAR/RXR signaling that goes beyond general descriptions. Additionally, the active RXR could also be heterodimerized with other transcription factors such as peroxisome proliferator‐activated receptors (PPARs) to regulate lipid metabolism [72], which is relevant to the physiological coupling between hepatopancreatic lipid and carotenoid deposition. Retinal can also be reduced by retinal reductase to form retinol, also known as vitamin A, the storage form through which carotenoid‐derived retinoid supply is buffered [93, 94]. Consistent with this metabolic role, vitamin A deficiency reduces survival in *Penaeus japonicus* and growth in *E. sinensis* [95], whereas sufficient vitamin A increases the height and surface area of intestinal villi and improves intestinal function [96]. These observations are best interpreted as downstream consequences of carotenoid‐to‐retinoid conversion efficiency rather than as independent vitamin A effects. Vitamin A is likely associated with the PI3K/Akt and AMP‐activated protein kinase (AMPK) pathways and consequently regulates protein translation, lipid and nucleotide synthesis, proliferation, and autophagy via the mTOR pathway [97, 98]. The transcriptions of mTOR and S6K1 in the hepatopancreas of *Procambarus clarkii* were significantly increased with sufficient vitamin A addition, while a decrease was found in 4E‐BP1, which binds to eukaryotic initiation factor eIF4E and inhibits ribosome recruitment to the mRNA [99].

Carotenoids and their retinoid derivatives further regulate oxidative and immune status of aquatic animals. Moderate reactive oxygen species (ROS) levels are physiologically necessary for pathogen clearance, whereas excess ROS cause DNA damage and apoptosis. Appropriate ROS can mediate antioxidative enzyme activity directly and trigger the nuclear factor E2‐related factor 2 (Nrf2) and MAPK/NF‐κB signaling pathways to control the immune response [100, 101]. It should be noted that dietary vitamin A can significantly improve the activities of acid phosphatase and alkaline phosphatase in *E. sinensis*, and the activated Nrf2 factor is capable of upregulating superoxide dismutase and catalase [95]. Toll‐like receptors (TLRs) are the most important receptors for innate immunity in crustaceans [102, 103]. The adaptor MyD88 activates NF‐κB by binding to TLRs and triggers the release of proinflammatory cytokines [104]. Astaxanthin plays an anti‐inflammatory role by regulating Toll‐like receptor 4 (TLR4) and the NF‐κB and MAPK signaling pathways in *Micropterus salmoides* and *Channa argus* [105, 106]. Additionally, TLRs also directly trigger the activation of the MAPK signaling pathway [107]. Although the mechanisms by which carotenoid participate in immunity remain to be elucidated, these findings collectively indicate an important role of retinoids in this process [108]. To maintain retinoid homeostasis, retinol tends to be esterified with fatty acyl groups present in the acyl‐CoA pool [72]. The lecithin:retinol acyltransferase is responsible for retinyl ester formation in retinoid visual cycle [109], whereas acyl‐CoA:retinol acyltransferase may be physiologically important in depositing retinyl esters [110]. Diacylglycerol acyltransferase for triglyceride synthesis may also act in the formation of retinyl esters [111]. Retinyl esters must be hydrolyzed to retinol before conversion to retinoic acid. Retinyl ester hydrolases are dedicated catalysts, and several enzymes have been proposed to release free retinol from retinyl ester pool [112]. Future work integrating enzyme‐level characterization with in vivo functional validation will be needed to fully define how dietary carotenoid intake is translated into these physiological outcomes.

## 3. Future Perspectives and Conclusions

This review systematically elucidates the key roles, metabolic pathways, and potential mechanisms of carotenoids in crab aquaculture. Crabs rely entirely on dietary intake of carotenoids, which regulate not only pigmentation but also growth, ovarian development, immunity, and antioxidant defense. Their selective deposition and metabolism in the hepatopancreas, ovary, and carapace directly impact individual health, reproduction, and ultimate market value. Carotenoid assimilation, including ingestion, transition, deposition, and transformation, is a complex and finely regulated biological process in crabs. The discovery of key metabolic enzymes and carrier proteins is revealing the underlying regulatory network; however, significant knowledge gaps remain regarding transport mechanisms in hemolymph, the regulation of tissue distribution, and the physiological functions of different optical and geometric isomers.

With the growing demand for sustainable aquaculture, future breakthroughs should focus on implementing precision feeding strategies and optimizing nutritional feed formulation, with environmental factors considered as key priorities. Further studies are required to determine the optimal carotenoid combinations, inclusion levels, and feeding duration for *E. sinensis*. Notably, metabolic and nutritional observations for marine farmed crabs (*e.g*., *P. trituberculatus* and *Scylla* spp.) remain scarce, and these management measures should be extrapolated to other crab species with caution. Given that natural carotenoid extraction from plant sources generally has limitations in cost efficiency and sustainability, further advances in synthetic biology are needed to construct biosynthetic pathways in noncarotenogenic microbes. A feasible development pathway would include construction of microbial host chassis with enhanced *β*‐carotene supply, screening and balancing of *β*‐carotene hydroxylase and ketolase modules, engineering of lipid droplets and esterification reactions to generate either free or esterified carotenoids (e.g., astaxanthin), and tailoring geometric and optical isomer compositions. The precise supply of carotenoids with specific configurations could significantly improve crab growth, coloration, and health. In addition, a deeper understanding of carotenoid absorption, transport, and tissue deposition is required. This will drive the design of precise nutritional strategies for specific physiological stages and health needs, thereby enhancing value across the entire chain from nutrient source to farmed crab products.

## Author Contributions

Chonglong Wang conceived the idea and refined the manuscript. Saeed Ullah and Muhammad Nadeem Khan investigated the topic and drafted the manuscript. Saeed Ullah, Muhammad Nadeem Khan, and Jiangtong Han performed data analysis; Jiangtong Han and Gongyuan Wei edited the manuscript and provided critical comments.

## Funding

This work was supported by the National Natural Science Foundation of China (Grant 21878198).

## Disclosure

All authors approved the publication of this work.

## Ethics Statement

This review did not involve any wet‐lab experiments with animals, and no ethical approval was required, following consultation with the Ethics Committee of Soochow University (ECSU), Suzhou.

## Conflicts of Interest

The authors declare no conflicts of interest.

## Data Availability

Data sharing is not applicable to this article as no datasets were generated or analyzed during the current study.
